# DIA-KARLOTTA (Kids + Adolescents Research Learning On Tablet Teaching Aachen): A Serious Game for Diabetes

**DOI:** 10.1177/19322968231194647

**Published:** 2023-08-30

**Authors:** Angeliki Pappa, Maria Tincheva, Lukas Menze, Laura Bell, Martin Lemos

**Affiliations:** 1 Department of Pediatrics, University Hospital RWTH Aachen, Aachen, Germany; 2 Medical Faculty, Audiovisual Media Center, RWTH Aachen University, Aachen, Germany

**Keywords:** pediatric diabetes, serious games, digital knowledge-test, transition-readiness, diabetes educational programs

“Education to protect tomorrow” was not only the motto of the *World Diabetes Day* 2022, but it is also our mission in the care of children and adolescents with type 1 diabetes (T1D). With over 18 000 youths diagnosed annually the need for educational programs to improve disease knowledge and self-efficacy in therapy implementation is paramount. Implementing these programs in the clinical routine should lead to enhanced therapy adherence and to improved transition-readiness.^1,2^ When asked for their wishes, children and adolescents demand communication and entertainment.^3^ Therefore, we developed a serious game web app, called KARLOTTA (Kids + Adolescents Research Learning On Tablet Teaching Aachen), in our clinic for children and adolescents with chronic diseases like T1D and inflammatory bowel disease (IBD) to facilitate individualized teaching. Before their appointment at our outpatient clinic, patients can utilize our web app, which includes short quizzes and mini games. Patients get immediate visual feedback about their correct or incorrect answers, and caregivers the results for further analysis. A previous version of the app has already been successfully tested in a randomized controlled study with 30 IBD patients.^4^

The aim of our current pilot study was to assess the acceptance as well as the functionality of the web app in the clinical routine and to collect qualitative feedback of T1D patients to improve user experience. Our quiz is based upon established educational programs used in Germany, Content: general knowledge T1D, nutrition, self-control, therapy—management, transition (>16 years).

A total of 8 patients took part in the evaluation (4 female, 3 male, 1 diverse). The mean age of the patients was 12.75 years (*SD* = 3.77). Their mean HbA1c level was 7.28 (*SD* = 0.89) and the mean duration of diabetes was 5.58 (*SD* = 1.53) years. All patients used a continuous glucose monitoring system (CGM), and while 7 patients had an insulin pump, only 1 had multiple daily injection therapy. The youngest participant (8 years) was excluded as certain questions were misunderstood. The patients evaluated the app using 3 types of questionnaires.

**Figure 1.**
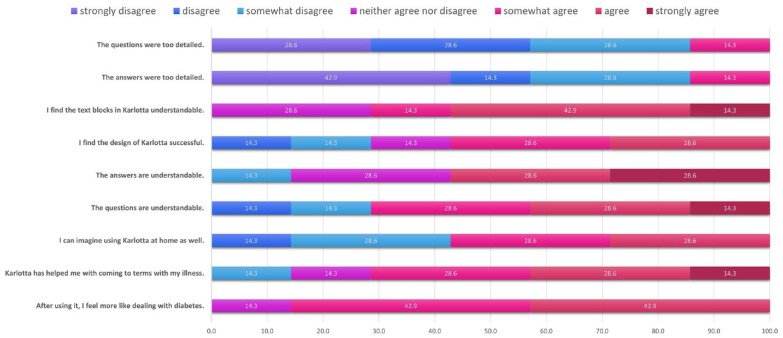
Results of questionnaire 3: Percentage (on the x-axis) of responses chosen by the patients per item.

We used the System Usability Scale (SUS)^5^ to measure the usability of the application. The resulting SUS acceptability was 76.80 (*SD* = 10.57). A value above 71.10 is considered “good.” Furthermore, the patients rated learning development, design, and appearance as good (2.5 out of 7; adaptation of the UXKQ^6^). The third questionnaire concerned motivation to deal with T1D and the difficulty of questions and answers. The results of this questionnaire are presented in Figure 1.

There is a need for creative solutions to guide and incentivize self-management motivation in T1D patients. Caregivers should be aware of the development in technology and use these resources for age-appropriate communication about sensitive issues and treatment of children and adolescents with T1D. Based on our experience, we conclude that our app KARLOTTA can be a useful tool in diabetes education and communication.

We are planning further prospective studies integrating knowledge quizzes, transition-readiness tests, and mini-games. By introducing more features, such as instructional videos, personalized avatars, and a feedback section we aim to enhance the interactive user experience.
